# Infective Endocarditis due to Esophageal Squamous Cell Carcinoma Invasion of the Left Atrium: A Case Report

**DOI:** 10.70352/scrj.cr.25-0568

**Published:** 2026-01-06

**Authors:** Takeru Nakamura, Ikuko Shibasaki, Masanobu Nakajima, Kazuyuki Ishida, Masatoshi Nakagawa, Kazuyuki Kojima, Hirotsugu Fukuda

**Affiliations:** 1 Department of Cardiac and Vascular Surgery, Dokkyo Medical University School of Medicine, Shimotsuga-gun, Tochigi, Japan; 2 Department of Upper Gastrointestinal Surgery, Dokkyo Medical University, Shimotsuga-gun, Tochigi, Japan; 3 Department of Diagnostic Pathology, Dokkyo Medical University, Shimotsuga-gun, Tochigi, Japan

**Keywords:** esophageal squamous cell carcinoma, infective endocarditis, direct invasion of the left atrium, cardiac involvement

## Abstract

**INTRODUCTION:**

Esophageal cancer is often diagnosed at an advanced stage and with distant metastases. While cardiac metastases from solid tumors have been reported in 0.2%–6.5% of postmortem cases, solitary cardiac metastases remain exceedingly rare. Infective endocarditis, a life-threatening condition typically associated with valvular involvement and bacteremia, has not been widely associated with cardiac metastases. Here, we present an extremely rare case of esophageal squamous cell carcinoma directly invading the left atrium during chemotherapy, which was further complicated by infective endocarditis.

**CASE PRESENTATION:**

A 52-year-old female who presented with chest and back pain and progressive dysphagia was diagnosed with esophageal squamous cell carcinoma following an endoscopic biopsy. Chemotherapy and chemoradiotherapy resulted in inadequate tumor control. During the second course of second-line chemoimmunotherapy, the patient developed a high fever, followed by seizures and loss of consciousness, and required emergency intubation. Echocardiography revealed a mobile mass 5 × 30 mm in size attached to the posterior wall of the left atrium. To prevent further embolic complications, emergency surgery was performed, which revealed a tumor invading the left atrial wall. Histopathological findings revealed a necrotic mass containing keratinizing squamous cell carcinoma fragments and a septic thrombus, consistent with infective endocarditis originating from the tumor surface. The patient did not regain consciousness following surgery, possibly because of hypoxic–ischemic brain injury following seizure-induced hypoxemia. Infection control remained poor, and the patient died of sepsis on POD 56.

**CONCLUSIONS:**

Direct invasion of the left atrium by esophageal squamous cell carcinoma is rare, and infective endocarditis arising from tumor surfaces within the cardiac chamber is exceptionally uncommon, particularly in immunosuppressed patients. In this case, surgery was performed to prevent further embolic events and remove the infected tumor mass. Although the direct source of the infection was surgically removed, infection control remained difficult, likely due to persistent infection under immunosuppressive conditions and the use of cardiopulmonary bypass. This case highlights the challenges of managing infective endocarditis in patients with cancer and suggests that early surgical intervention may help reduce embolic risk, even when complete infection control cannot be achieved.

## Abbreviations

AC
: adenocarcinoma
CF-PEM
: cisplatin, 5-fluorouracil, plus pembrolizumab
CPB
: cardiopulmonary bypass
CRT
: chemoradiotherapy
DCF
: docetaxel, cisplatin, and 5-fluorouracil
EGD
: esophagogastroduodenoscopy
FDG
: 18F-fluorodeoxyglucose
IE
: infective endocarditis
LA
: left atrium
SCC
: squamous cell carcinoma
TTE
: transthoracic echocardiography

## INTRODUCTION

Esophageal cancer, a highly lethal malignancy, is typically diagnosed at an advanced stage, after the development of distant metastases.^1)^ In de novo Stage IV disease, the predominant histological subtype determines the metastatic pattern: SCC frequently metastasizes to the lungs, whereas AC more commonly spreads to the liver and brain. The incidence of bone and distant lymph node metastases, while high, does not differ significantly between the SCC and AC subtypes.^1)^

Cardiac metastases from malignant tumors are relatively rare, with reported incidences of 2.3%–18.3% in patients with cancer and 0.2%–6.5% in postmortem cases. Solitary cardiac metastases are particularly uncommon.^2)^

IE most commonly occurs in the four weeks following invasive dental procedures (odds ratio: 2.00; 95% confidence interval: 1.59–2.52)^3)^; IE caused by cardiac tumors is extremely rare.

Here, we report an extremely rare case of esophageal SCC directly invading the LA during chemotherapy, which was further complicated by IE.

## CASE PRESENTATION

**Fig. 1:**
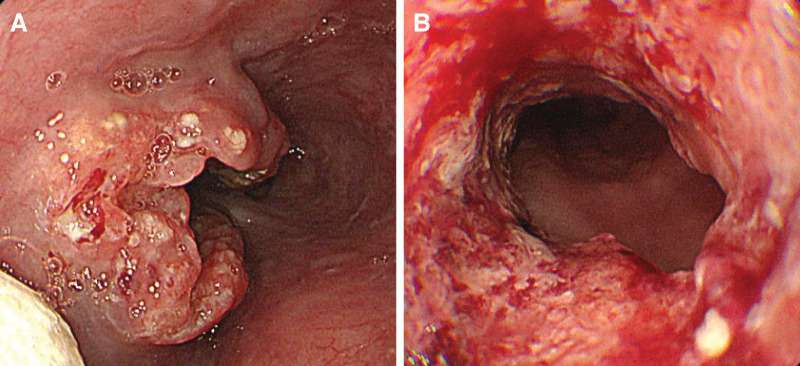
Esophagogastroduodenoscopy findings. (**A**) A type 2 tumor in the middle thoracic esophagus. The white object in the foreground is the remains of a pill that was ingested. (**B**) The center of the tumor is circumferential.

**Fig. 2:**
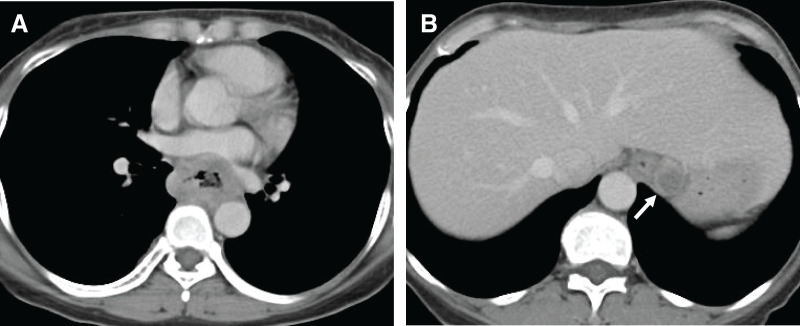
CT images. (**A**) A large tumor in the middle thoracic esophagus compresses the heart; invasion of the aorta is suspected. (**B**) A swollen left paracardial lymph node (arrow).

**Fig. 3:**
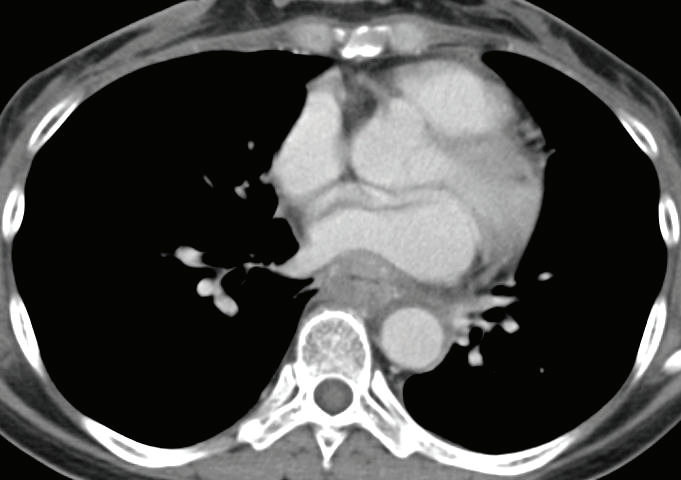
CT image. An esophageal primary esophageal tumor is compressing the left atrium, but direct invasion is not evident.

**Fig. 4:**
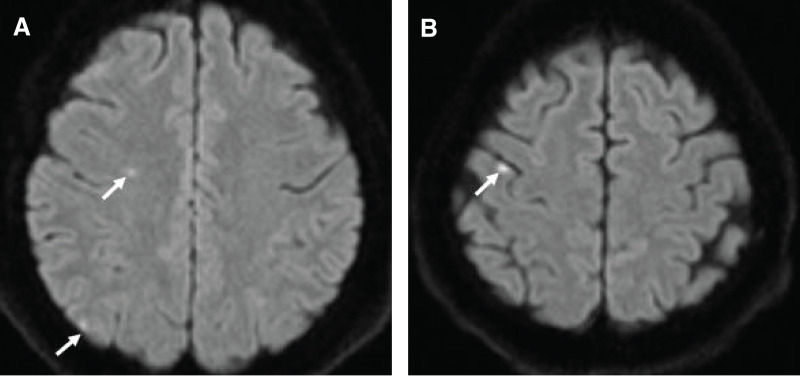
MRI findings. (**A**, **B**) Diffusion-weighted imaging showing scattered high signals in the right cerebral hemisphere (arrows).

**Fig. 5:**
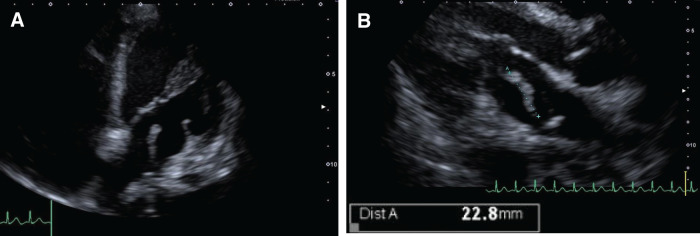
Transthoracic echocardiography findings. (**A**) A mobile mass is attached to the posterior wall of the left atrium. (**B**) The mass has a length of approximately 22.8 mm.

A 52-year-old female presented to a local doctor complaining of chest and back pain and dysphagia. An EGD revealed a circumferential tumor in the middle thoracic esophagus, and the patient was referred to our hospital with a diagnosis of esophageal cancer. A further EGD was performed at the Department of Upper Gastrointestinal Surgery (**Fig. 1**), and SCC was detected by biopsy. Contrast-enhanced CT revealed suspected tumor invasion of the descending aorta (**Fig. 2A**) and left paracardial lymph node swelling (**Fig. 2B**). FDG PET revealed FDG uptake in the primary tumor and left paracardial lymph node. The patient was diagnosed with esophageal cancer T3br(aorta)N1M0 Stage IIIB,^4,5)^ and induction chemotherapy with DCF was initiated in accordance with department policy.^6)^ No reduction in the tumor was observed following one course of DCF therapy, and dysphagia worsened; therefore, definitive CRT was performed, again using DCF. As CRT resulted only in stable disease, CF-PEM therapy was initiated. The first course of CF-PEM therapy was completed without complications. However, the patient developed a high fever immediately after starting the second course. A contrast-enhanced CT scan was performed on the same day. The primary esophageal tumor was compressing the LA, but there were no findings strongly suggestive of direct invasion, and no air in the atrium, which would suggest an atrio-esophageal fistula, was observed (**Fig. 3**). TTE performed on the same day also failed to confirm direct invasion of the LA. The condition of the patient suddenly deteriorated the following evening, as evidenced by convulsions and loss of consciousness. Brain MRI revealed a cerebral infarction (**Fig. 4**). Spontaneous breathing was present but insufficient to maintain oxygenation; emergency intubation was required. TTE performed the following day to identify the embolic source revealed a mobile, cord-like structure approximately 22.8 mm in length protruding from the posterior wall of the LA, indicative of a cardiac tumor (**Fig. 5**).

*Streptococcus anginosus, Streptococcus mitis/oralis*, and *Klebsiella pneumoniae* were cultured from blood samples, raising concerns about IE associated with vegetation.

The patient exhibited embolic symptoms due to the mobile mass or vegetation arising from the posterior wall of the LA. The infection remained uncontrolled despite the administration of appropriate antibiotic therapy. As a result of the TTE findings, the patient was referred to our department for further management. Although the level of consciousness of the patient could not be fully assessed, considering her relatively young age and the strong wishes of her family, the decision was made to perform emergency surgical resection of the suspected tumor or vegetation on the day following the onset of cerebral infarction, despite the poor prognosis.

### Surgical procedure

Following median sternotomy, CPB was performed using arterial cannulation via the ascending aorta and bicaval venous cannulation.

A vertical approach to the LA revealed a pedunculated tumor-like mass arising from its posterior wall. The mass was approximately 5 × 30 mm in size; a thrombus was observed on its stalk.

The tumor was easily removed with gentle traction using forceps, leaving a small defect approximately 5 mm in size at the base. The defect was closed with two 4-0 Prolene sutures and an autologous pericardial felt strip, followed by patch closure with a 30 × 30-mm autologous pericardial patch and a continuous 5-0 Prolene suture. Careful inspection of the pericardial cavity revealed no metastatic lesions.

The surgical procedure was recorded on video (**Supplementary Video**).

The aortic cross-clamp time was 61 min, the CPB time was 85 min, and the total operative time was 219 min.

### Histopathological findings

**Fig. 6:**
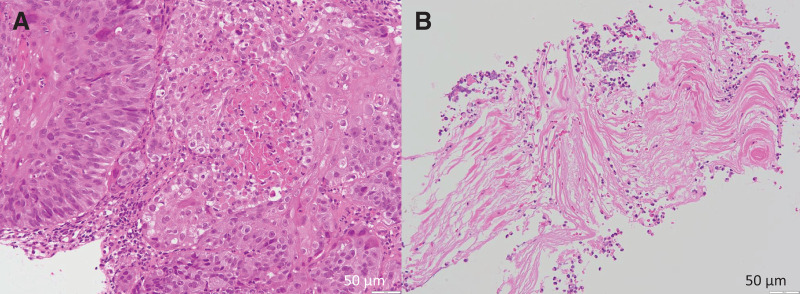
Histopathological findings. (**A**) Histopathology of the esophageal biopsy reveals an invasive keratinizing squamous cell carcinoma. (**B**) Squamous cell carcinoma-derived keratin plaque in the resected left atrial mass. Sections were stained with hematoxylin and eosin. Magnification, ×200. Scale bar = 50 μm.

Histopathology of the initial esophageal tumor biopsy, obtained during the EGD, revealed a keratinizing SCC (**Fig. 6A**) with atypical squamous cells invading the subepithelial stroma.

Histopathology of the resected LA mass showed that it predominantly consisted of necrotic fibrin and neutrophilic aggregates, suggestive of IE. Keratin plaques, which were presumed to have originated from the esophageal carcinoma, were embedded in the tissue fragments (**Fig. 6B**). No viable tumor cells were observed.

### Postoperative progress

The patient did not regain consciousness following surgery. Although the exact reason for this was unclear, hypoxic–ischemic brain injury associated with seizure-induced hypoxemia and multiple embolic infarctions may have been contributing factors. Although the infected cardiac mass was successfully removed, infection control remained difficult, possibly due to chemotherapy-induced immunosuppression, persistent bacteremia before surgery, and the additional immunological burden associated with CPB; the patient died of sepsis on POD 56.

## DISCUSSION

This case highlights 3 important clinical points. First, although exceptionally rare, advanced esophageal cancer can directly invade the LA, resulting in intracardiac extension. Second, IE in immunocompromised patients with cancer may involve not only the native valves but also the tumor surfaces, suggesting that the malignant tissue itself can serve as a nidus for infection. Third, surgical resection through open-heart surgery plays a crucial role in infection control and the prevention of embolic complications.

IE is a life-threatening disease. Although relatively uncommon, its incidence and mortality have increased steadily over the past three decades.^7)^ IE typically affects native or prosthetic valves or is associated with intracardiac devices; cardiac tumors rarely become the focus of infection.^8)^ The growing use of implantable cardiovascular electronic devices has contributed to the rising incidence of device-related IE, which now accounts for 20%–25% of all device-associated infections.^9)^

In high-risk populations such as individuals with a history of IE, prosthetic valves, or congenital heart disease, a significant temporal association has been observed between IE onset and invasive dental procedures conducted within the preceding four weeks. Antibiotic prophylaxis has been shown to significantly reduce IE incidence in these individuals (odds ratio: 0.49; 95% confidence interval: 0.29–0.85).^3)^

IE typically involves the cardiac valves, where vegetation forms on the endocardial surface and may lead to perivalvular abscess formation.^8)^ In contrast, the vegetation in the present case was observed on the posterior wall of the LA, at the site of direct tumor infiltration, a highly unusual anatomical location for IE development.

To date, only 17 cases of cardiac involvement in living patients with esophageal cancer have been reported, including the present case; approximately 70% of these patients presented with symptoms or clinical signs.^10,11)^ Cardiac metastasis typically occurs via hematogenous or lymphatic spread.^2)^ Including the present case, a total of 13 cases of SCC with cardiac involvement have been reported. Most were hematogenous in origin; direct invasion of the left atrium, as observed in the present case, is exceedingly rare.^10,12–22)^ Unlike prior reports, which have mainly described metastatic deposits in the right ventricle or atrium, our case demonstrates direct extension of the primary tumor into the left atrial cavity. Although aortic invasion was initially suspected in this case, our limited experience of direct LA invasion led to inadequate predictions. For cancers located in the mid-thoracic esophagus and with a strong tendency to invade, such as macroscopic type 3 cancers that are in contact with the heart, it is important to regularly check for invasion using high-resolution CT and TTE. In the event of a sudden fever, it is important to perform a prompt examination, considering the possibility of direct invasion of the heart. The common oral commensals *S. mitis/oralis* and *K. pneumoniae* were cultured from the blood of the patient in the present case. The patient had no recent history of dental treatment, rendering hematogenous spread from the oral cavity unlikely. Histological analysis revealed fragments of SCC directly invading the LA, suggesting that bacterial translocation may have occurred via mucosal breakdown caused by ulcerative esophagitis. Notably, the infection occurred during immunosuppressive chemotherapy. During chemotherapy performed prior to the onset of IE, the white blood cell and neutrophil counts of the patient were 3400–3900 and 2500–2900/μL, respectively. Based on reference values, it cannot be said with certainty that the patient was immunosuppressed. However, the white blood cell count had declined from approximately 8000/μL before the first course of chemotherapy, and so the possibility that this patient was chronically immunocompromised cannot be ruled out. The presence of septic thrombi within the cardiac tumor further supported the diagnosis of tumor surface-associated IE leading to sepsis.

The key surgical objective in this case was to remove the infectious source while minimizing operative time and surgical trauma. Because the defect at the base of the tumor was approximately 5 mm in diameter, and the patient was relatively young, firm closure was required to prevent postoperative bleeding and infection recurrence. Therefore, patch closure was performed using an autologous pericardial patch to ensure a secure and durable repair. The aortic cross-clamp time of 61 min was within a reasonable range, and the procedure was completed efficiently. This approach was considered appropriate to achieve a reliable repair and reduce the risk of recurrence.

## CONCLUSIONS

Direct invasion of the left atrium by esophageal SCC is rare, and IE arising from tumor surfaces within the cardiac chamber is exceptionally uncommon, particularly in immunosuppressed patients. In this case, surgery was performed to prevent further embolic complications and remove the infected tumor mass. Although the direct source of infection was surgically removed, infection control remained difficult, likely due to persistent infection under immunosuppressive conditions and the use of CPB. This case highlights the challenges of managing IE in patients with cancer and suggests that early surgical intervention may help reduce embolic risk, even if infection control remains difficult.

## SUPPLEMENTARY MATERIALS

Supplementary Video

## DECLARATIONS

### Availability of data and materials

The datasets supporting the conclusions of this article are included within the article.
